# Contribution of Inhibitor of DNA Binding/Differentiation-3 and Endocrine Disrupting Chemicals to Pathophysiological Aspects of Chronic Disease

**DOI:** 10.1155/2017/6307109

**Published:** 2017-07-13

**Authors:** Vincent Avecilla, Mayur Doke, Quentin Felty

**Affiliations:** Department of Environmental & Occupational Health, Florida International University, Miami, FL, USA

## Abstract

The overwhelming increase in the global incidence of obesity and its associated complications such as insulin resistance, atherosclerosis, pulmonary disease, and degenerative disorders including dementia constitutes a serious public health problem. The Inhibitor of DNA Binding/Differentiation-3 (ID3), a member of the ID family of transcriptional regulators, has been shown to play a role in adipogenesis and therefore ID3 may influence obesity and metabolic health in response to environmental factors. This review will highlight the current understanding of how ID3 may contribute to complex chronic diseases via metabolic perturbations. Based on the increasing number of reports that suggest chronic exposure to and accumulation of endocrine disrupting chemicals (EDCs) within the human body are associated with metabolic disorders, we will also consider the impact of these chemicals on ID3. Improved understanding of the ID3 pathways by which exposure to EDCs can potentiate complex chronic diseases in populations with metabolic disorders (obesity, metabolic syndrome, and glucose intolerance) will likely provide useful knowledge in the prevention and control of complex chronic diseases associated with exposure to environmental pollutants.

## 1. Introduction

Inhibitor of DNA Binding/Differentiation-3 is a member of the ID family of helix-loop-helix proteins encoded by an immediate-early gene responsive to mitogenic signals and oxidative stress. ID3 functions as a transcriptional regulator known to prevent stem cell differentiation and promote cell cycle progression. An increasing body of evidence suggests that ID3 may be involved in metabolic perturbations characterized by insulin resistance, hyperglycemia, abdominal obesity, dyslipidemia, and hypertension. Interactions across multiple organ systems that contribute to metabolic perturbations present a challenge to ongoing research attempting to elucidate biological mechanisms of chronic disease associated with metabolic health. For instance, insulin resistance and systemic low-grade inflammation result from complex interactions between the vasculature, metabolic tissue, and immune cells. With regard to these interactions, it is noteworthy that ID3 plays a significant role in vasculogenesis, energy metabolism, and development of the immune system. In the vasculature, ID3 is essential to embryonic vasculogenesis and endothelial cell activation [1–3]. Given that metabolic perturbations are observed in endothelial cells from diseased vasculature [4], ID3 may mediate endothelial dysfunction often found in individuals with metabolic syndrome. In addition to the vasculature, ID3 function spans to metabolic tissue and immune cells. In vivo studies have demonstrated that ID3 mediates high fat diet-induced obesity and promotes obesity-induced inflammatory macrophage accumulation [5, 6]. Thus, we intend to discuss the current understanding of how ID3 may influence chronic diseases associated with metabolic perturbations. Since adipose tissue is an endocrine organ as well as a metabolic organ, exposure to endocrine disrupting chemicals (EDCs) may also contribute to metabolic perturbations associated with chronic disease. EDCs are mostly synthetic chemicals ubiquitously found in our environment that act by altering hormone action. Estrogenic EDCs such as diethylstilbestrol (DES), bisphenol A (BPA), estrogenic polychlorinated biphenyls (PCBs), and phthalates have been implicated to interfere with metabolic health during critical periods of development and into adulthood. Epidemiological studies have reported associations between exposure to EDCs and metabolic syndrome [7–9]. Based on our recent findings that showed ID3 dependent endothelial cell activation by exposure to estrogenic PCB congener 153 [10, 11], we will also discuss how low-dose EDC exposure from the environment may potentiate complex chronic disease in populations with metabolic disorders (obesity, metabolic syndrome, and glucose intolerance) via ID3. A better understanding of interactions between ID3 and EDC is critical in deepening our understanding of how environmental factors modify chronic disease risk and health outcomes. Further study in these areas may reveal novel or more effective therapeutic modalities as well as provide prevention and control strategies of complex chronic diseases associated with exposure to EDCs.

## 2. Inhibitor of DNA Binding/Differentiation-3 (ID3)

### 2.1. Structure and Function

The ID (Inhibitor of DNA Binding/Differentiation) family of small proteins consists of four genes (ID1-ID4). The four members of the ID family share extensive amino acid sequence homology (69–78%) within their helix-loop-helix (HLH) domain [12, 13], but the remaining parts of the proteins are nonrelated. Experimental studies in genetically engineered mice have revealed the importance of ID3 in embryonic development and cell differentiation. ID3 gene knockout mice are viable; however, they have demonstrated defects in immune cell differentiation [14, 15]. In contrast, double ID1/ID3 knockout mice showed abnormal vascularization of the brain [16], neuronal differentiation, and cardiac defects [17] that were embryonically lethal. Resistance to tumor angiogenesis was reported in mice deficient in 1–3 alleles of ID1/ID3 gene knockout combination [12]. ID3 is highly expressed in embryonic tissue but declines as cells differentiate [12]. In adult tissues, the expression of ID3 is context specific and tends to be highest in proliferating and undifferentiated cells. ID3 expression has been reported to be induced by diverse stimuli in many cell types [18].

**Figure 1:**
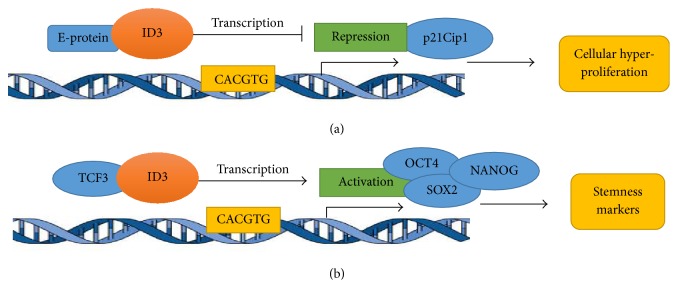
ID3 transcriptional regulation. Scheme illustrating how ID3 can repress expression of p21 gene (a) or activate gene expression of embryonic transcription factors (b).

The ID3 gene was initially identified as a serum-inducible immediate-early gene in mouse fibroblasts that peaks transcriptionally at 1 h [14, 19]. Subsequently, ID3 expression has been reported to be biphasic with maximal stimulation at 1 h following a second burst at 24 h as in the case of tissue regeneration after injury. We and others have shown that ID3 expression is redox sensitive [1, 20]. Specifically, we have shown that vascular endothelial cells exposed to either 17*β*-estradiol (E2) or the estrogenic PCB congener 153 (PCB153) resulted in increased ID3 expression, protein phosphorylation, and endothelial neovascularization. Treatment with reactive oxygen species scavengers inhibited estrogenic chemical induced neovascularization [10, 21]. Proteasomes reportedly degrade ID3 by an ubiquitin dependent mechanism. The protein half-life of ID3 has been demonstrated to be approximately 20 min in HEK293 cells [13]. In mammals, ID protein-protein interactions occur via the HLH motif in which ID proteins dimerize and block the DNA binding activity of basic HLH transcription factors, such as a group of E proteins (E12/E47, E2-2, and HEB) encoded by the TCF3, TCF4, and TCF12 gene, respectively. Among these E proteins, ID3 has been most often reported to interact with E12/E47 [22]. The E proteins are basic HLH transcription factors that bind to the E-box consensus sequence (CANNTG) in the promoter of target genes. ID3 plays an important role in cell proliferation via its interactions with E proteins. For example, E proteins have been shown to bind the E-box sequence in the promoter of the cyclin dependent kinase inhibitor p21^Cip1^ and activate its transcription [23]. The level of p21^Cip1^ is elevated in quiescent cells where it acts as a suppressor of cell proliferation [24]. In the context of the cell cycle, ID3 promotes cell cycle progression by the inhibition of p21^Cip1^ expression [25]. Specifically, ID3 protein-protein interactions with E proteins can disrupt their ability to bind gene promoters and thereby block transcriptional activation by these factors. ID3 has been shown to inhibit E proteins from activating the p21^Cip1^ promoter in proliferating vascular cells [26]. Thus, ID3 has been frequently described as a dominant negative inhibitor of E proteins. Although ID proteins have been shown to function as dominant negative transcriptional regulators of E proteins, there may be circumstances by which ID3 acts as a positive transcriptional regulator. ID3 has been shown to regulate the binding of transcription factor 3 (TCF3) to the E-box motif in target gene promoters [27]. TCF3 has been reported to repress the expression of pluripotency genes OCT4, SOX2, and NANOG that contribute to cell differentiation [14]. Our research has shown that ectopic overexpression of ID3 increased OCT4 and SOX2 expression in endothelial cells and resulted in a population of cells that were positive for the molecular stemness signature CD133^+^ VEGFR3^+^ CD34^+^ [28]. These endothelial stem cells were morphologically differentiated into smooth muscle cells and neuron cells. Based on these lines of evidence, ID3 maintains cells in an undifferentiated or noncommitted state by preventing the repression of pluripotency factors by TCF3. Hence, it is also plausible for ID3 to function as a positive regulator of gene transcription. In lieu of a recent report that showed ID3 to modulate genes essential for maintaining genome integrity during cell division [29], a dual regulatory role of ID3 in both positive and negative gene transcription expands its influence as shown in Figure 1. ID3 protein-protein interactions are not exclusive to E proteins as ID proteins have also been reported to bind to proteins that do not contain the HLH motif such as caveolin-1 [30].

### 2.2. ID3 and Metabolic Syndrome (MetS)

There has been increasing evidence that ID3 plays a role in adipogenesis. ID3 through adiponectin is considered to improve *β*-cell function, circulating lipids, and insulin sensitivity levels [31, 32]. ID3 inhibits transcriptional activity of E47 in undifferentiated preadipocytes [6]. ID3 negatively inhibits the FAS (fatty acid synthase) promoter via SREP-1c in adipose tissue. ID3 furthermore plays a role in blood glucose, which if dysregulated can lead to insulin resistance. In human islet cells ID1 and ID3 mRNA levels are increased with addition of glucose [33]. The induction of ID1 and ID3 expression, insulin secretion, and gene transcription suggests that IDs may play a role in promoting *β*-cell function [33, 34].

### 2.3. ID3 and Endocrine Disrupting Chemicals Influence Metabolic Perturbation

Metabolic syndrome (MetS) and its associated complications such as insulin resistance, abdominal obesity, dyslipidemia, and hypertension contribute to chronic diseases including cardiovascular disease (CVD), type 2 diabetes, cancer, and chronic kidney disease (CKD). Some studies have shown the prevalence of MetS in the United States at approximately 34% of the adult population [35]. MetS is an illness of energy consumption and storage which is a diagnosis of the cooccurrence of a minimum of three of the following medical conditions: abdominal obesity, high triglyceride levels, low HDL cholesterol levels, high fasting blood sugar, and high blood pressure. The molecular mechanisms of MetS are not fully understood. Most patients are older, sedentary, and obese and have a certain amount of insulin resistance. Important factors that are associated with MetS can include aging, diet, sedentary behavior, genetics, excessive alcohol use, or low physical activity [16, 36–38]. MetS appears to have three conceivable etiological groupings: obesity and disorders of adipose tissue; insulin resistance; and a collection of independent factors (e.g., molecules of hepatic, vascular, and immunologic origin).

Inflammatory factors produced during obesity are a major pathway for developing metabolic perturbation which can lead to MetS. Experimental studies have demonstrated ID3 to be a key regulator of monocyte chemoattractant protein-1 (MCP-1) [39]. MCP-1 is a well-known chemokine impacted by MetS [40]. ID3 has also been reported to regulate the production of interleukins IL-5, IL-6, IL-8, and IL-10 [41–43]. The induction of these chemokines has been observed in population studies of obesity and/or MetS. ID3 is also an oxidative stress regulated gene which may provide a positive feedback pathway in response to metabolic perturbations [1, 20]. Taken together, these lines of evidence provide the basis for how ID3 can participate in metabolic perturbations via controlling the expression of inflammatory factors involved in obesity and/or MetS. A growing number of reports implicate endocrine disrupting chemicals (EDCs) as an environmental factor that contributes to the occurrence of MetS. We performed a comprehensive search in the Comparative Toxicogenomic Database (CTD) to identify known ID3 and EDC interactions with results shown in Table 1 [44].

Adipose tissue is highly connected to steroid hormones (estrogens, androgens, and glucocorticoids) and maintains a close relationship with the immune system via adipokines. Endocrine disruption can interfere with the creation, discharge, breakdown, elimination, and imitation of natural hormones [45]. EDCs can be cataloged into multiple groupings such as dioxins, organotins, plastics, and pesticides. The increased presence of EDCs in the environment may help explain the incidence of metabolic disorders and associated complications. EDCs are found in everyday products (including food, plastic bottles, metal cans, toys, cosmetics, and pesticides) and used in the manufacture of food. Exposure to EDCs may regulate inflammatory factors via ID3. TCDD and PCB congeners have been shown to upregulate MCP-1 expression [46]. Bisphenol A exposure has been reported to increase IL-6 [47]. Population studies furthermore have reported an association between bisphenol A plasma levels and proinflammatory cytokines including IL-6 [48].

Besides inflammation, ID3 may contribute to other risk factors of MetS such as angiogenesis, adipose tissue, blood glucose levels, and insulin resistance. PCBs have been associated with MetS in epidemiological studies [49]. In the mouse model, PCB153 has been shown to produce significant metabolic changes when administered with a high fat diet that were consistent with worsened obesity and nonalcoholic fatty liver disease pathology [50]. ID3 may contribute to MetS via visceral fat expansion that was demonstrated in mice fed a high fat diet [5]. ID3 deficiency resulted in greater energy expenditure and higher metabolic rate in mice at rest. With respect to metabolic disorders involving high glucose levels, ID3 may be impacted because it was shown to be regulated by glucose in pancreatic islet *β*-cells [33] and under chronic hyperglycemic conditions. ID family proteins were stabilized which in turn activated metabolic genes [51]. Since a causal link of diabetes and vascular disease is chronic hyperglycemia, ID3 may also contribute to metabolic perturbations from high blood glucose levels. More importantly, however, mitochondrial reactive oxygen species (ROS) produced by vascular endothelial cells under hyperglycemic conditions may share a pathway similar to environmental toxicant induced oxidative stress which converges on ID3. PCBs have been reported to have estrogenic activity [52–55]. PCB153 is an agonist for the pregnane X receptor (PXR) and the constitutive androstane receptor (CAR) which exert their effects on energy metabolism through gene regulation [56]. Hence, dysregulation of these receptors by EDCs may contribute to PCB-induced metabolic perturbations. Several epidemiological studies have reported the association of PCB exposure with the increased risk of cardiovascular disease [57–61]. Since cardiovascular disease is a chronic disease affected by metabolic perturbations, we investigated the effects of PCB153 exposure on ID3 in vascular cells. Based on known PCB blood levels from occupational exposure, we showed a significant increase in PCB153-induced vascularization at doses of 10–100 ng/mL which was ID3 dependent [11]. We have shown that PCB-induced ROS mediated a highly branched neovascular phenotype depending on ID3 and Pyk2 [10]. Because the level of ID3 protein is determined by the rates of protein synthesis and protein degradation, we tested if PCB153 treatment affected ID3 protein synthesis. We showed that estrogenic chemical induced ID3 did not depend on protein synthesis; instead PCB153 treatment increased ID3 protein stability in endothelial cells. The role of phosphorylation in the regulation of ID3 protein stability is not known. A search with the PhosphoMotif finder program revealed that ID3 had 38 serine kinase/phosphatase motifs and 8 tyrosine kinase/phosphatase motifs [62]. We reported that both E2 and PCB153 induced ID3 phosphorylation. E2 treatment stabilized ID3 protein during the 1–6 h treatment time points and increased phosphorylated ID3 levels [10, 21]. It was noteworthy that we showed data of ID3 tyrosine phosphorylation by PCB153 treatment which was confirmed by MALDI-TOF MS/MS spectra data. Our findings revealed phosphorylated amino acids Tyr-11 and Tyr-48 in peptides from ID3. Interestingly, Tyr-48 is positioned in the helix-loop-helix motif that is essential for protein binding. Currently, it is not clear whether PCB153-induced phospho-ID3 leads to protein-protein interactions that may prevent its degradation; however, our findings demonstrated that ID3 is a target of posttranslational modifications by the endocrine disruptor PCB153 in vascular endothelial cells. Based on these lines of evidence, estrogenic chemical induced ID3 signaling contributes to hyperplastic vascular lesions. This vascular cell dysfunction may be a pathway by which EDCs and ID3 contribute to cardiovascular disease [1]. Based on these findings, we propose that ID3 is a target of EDCs that can activate inflammatory and energy pathways susceptible to metabolic perturbation during chronic disease pathogenesis. In the next section, we intend to discuss the current mechanistic understanding of how ID3 may influence chronic diseases associated with metabolic perturbations.

## 3. ID3 and Disease Outcomes

### 3.1. Vascular Diseases

**Figure 2:**
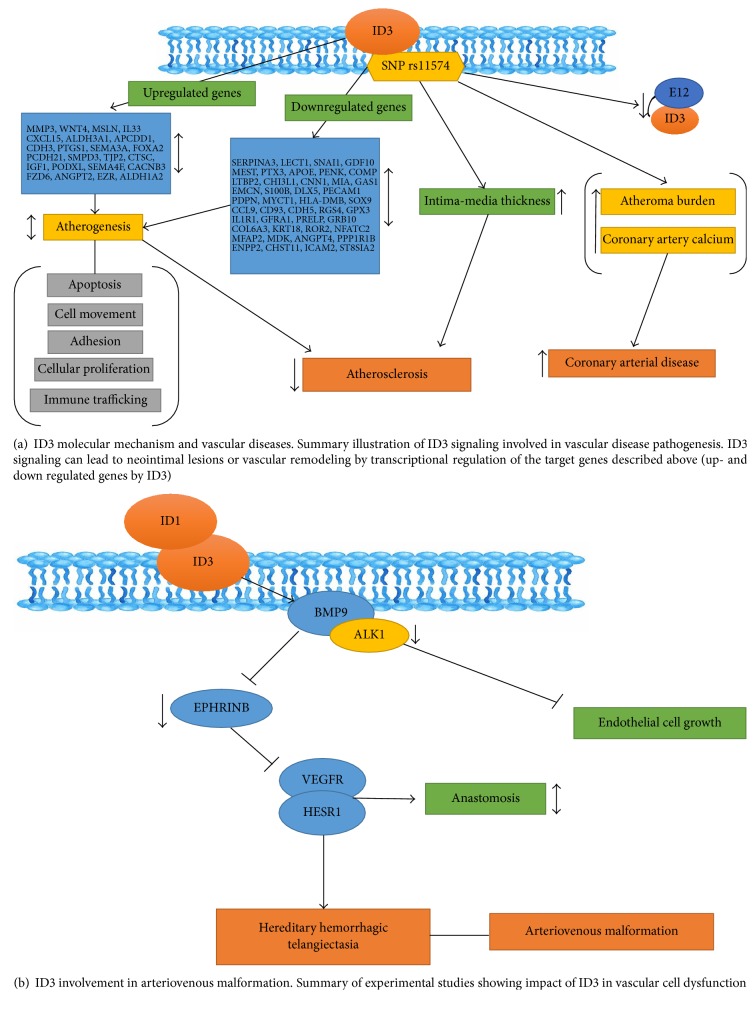


ID3 involvement in vascular disease has been studied together with the lipoxygenase (12/15-LO) which is known to generate proinflammatory changes in blood vessels that precede the development of atherosclerosis [63]. 12/15-LO is an important mediator of VSMC growth and its growth-promoting effects were shown to be mediated by ID3 transcription [64]. Increased expression of 12/15-LO in the vessel wall enhanced ID3-dependent cell proliferation, fibronectin deposition, and neointimal formation. Population-based studies have found SNP (single nucleotide polymorphisms) rs11574 in the coding region of the human ID3 gene associated with subclinical atherosclerosis in the Diabetes Heart Study [65]. ID3 SNP rs11574 showed a significant association of coronary artery disease for Caucasians and to a lesser extent African Americans and Hispanics [66]. Ectopic expression of ID3 in VSMC (vascular smooth muscle cells) regulates the cell cycle [67]. ID3 has also been shown to play a complex role with atherosclerosis. ID3 expression is increased by hyperlipidemia and oxidized LDL [26]. ID3 regulates angiotensin II and carotid intima-media thickness. Angiotensin II promotes hyperplasia through upregulating ID3 [68]. The ID3 SNP could be a potential loss of function mutation if it inhibits the functioning of E proteins, thus being an atheroprotective factor. As shown in Figure 2, ID3 may impact vascular cell dysfunction leading to intimal lesions.

ID3 stimulates visceral adipose VEGFA expression, depot expansion, and microvascular blood volume [5]. ID3 promotes angiogenesis in HFD- (high fat diet-) induced visceral adiposity [5]. ID3 KO shows a protective effect from HFD-induced visceral fat depot expansion. Furthermore, HFD-induced VEGFA expression in visceral adipose tissue was completely abolished by loss of ID3. BMP9 induces both ID1 and ID3 which are necessary for induction of Ephrin B2 [69]. A summary in Figure 2(b) shows an ID3 signaling pathway involved in vascular malformations.

### 3.2. Neurological Disorders

**Figure 3:**
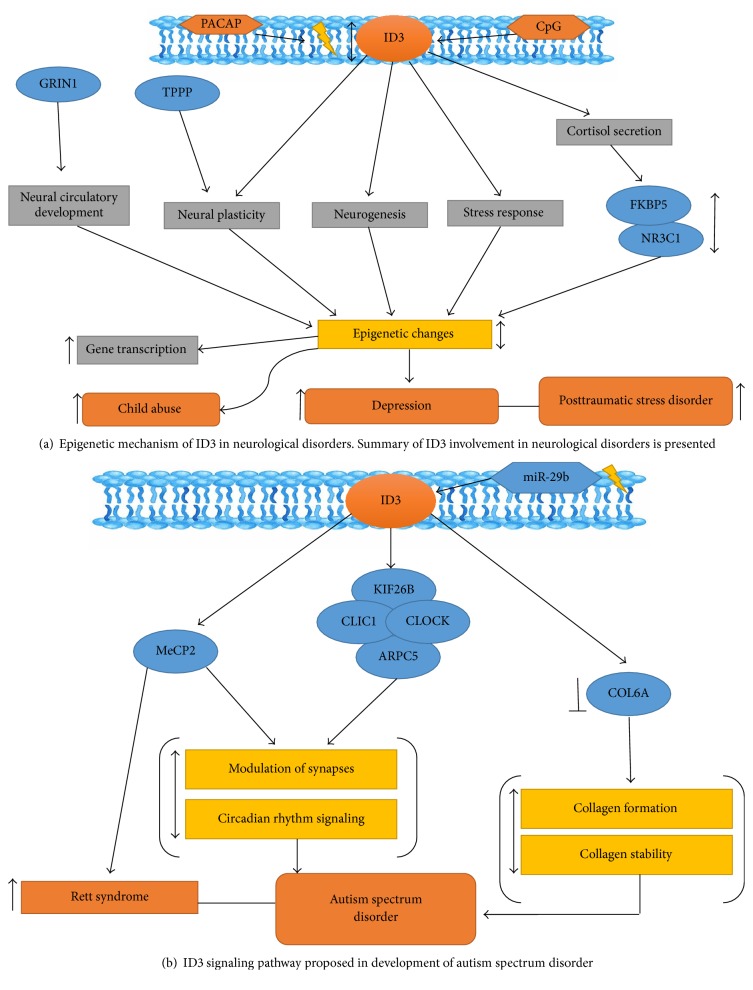


The ID3 gene is biologically relevant to neurological and behavior research because of its involvement in the stress response, neural plasticity, and neural circuitry. Molecular genetic studies in mice have shown that ID3 is required for embryonic neurovascular development. Genetic loss of ID1 and ID3 led to deviant neurovascular formations resulting in death [12]. ID1 and ID3 mutants showed premature differentiation of CNS radial glial cells that greatly increased neurons. Since radial glial cells function as scaffolds for developing blood vessels in the CNS, alterations to their development in ID1/ID3 knockout mice may contribute to deviant blood vessel morphogenesis and hemorrhage. ID1 and ID3 may function beyond maturation of the CNS neurovasculature because other pathways such as Notch1 activation do not support neurovascular disorders [70, 71]. Psychopathologies such as anxiety and depression have been associated with ID3 methylation status. Epigenetic changes in ID3 have been associated with maltreatment of children and demonstrated as a predictor for depression. Montalvo-Ortiz et al. reported epigenetic alterations in DNA derived from saliva in three genes predicted depression in a cohort of maltreated children: ID3, Glutamate NMDA Receptor (GRIN1), and Tubulin Polymerization Promoting Protein (TPPP) [72]. Studies of the expression of these genes from medial prefrontal cortex (mPFC) tissue of mice are subjected to a model of maternal neglect, which comprised maternal separation and early weaning (MSEW). Behavioral tests were performed in MSEW and control adult male mice by the higher plus maze (EPM) and forced swimming test (FST), respectively [72]. Behavioral differences in the EPM and FST tests showed that these genes, ID3, GRIN1, and TPPP, could predict anxiety and depression. Based on these studies, ID3 may contribute to the etiology of anxiety and depressive phenotypes when exposed to early life stress [72] (Figure 3(a)).

Both environmental and genetic factors contribute to the progression of MetS and neurodegenerative disorders [73]. Numerous studies have demonstrated that prediabetes and diabetes mellitus support cognitive decline related to Alzheimer's disease (AD) and vascular dementia [73]. For example, sucrose-treated mice develop mitochondrial abnormalities with significant increase in A*β* levels and slight increase in pTau levels which links metabolic perturbations from sucrose consumption with the AD-like pathology. Epigenetic changes in ID3 have been associated with maltreatment of children and demonstrated as a predictor for depression [74]. Another epigenetic study of individuals with autism spectrum disorders (ASD) revealed a significant association with a microRNA that targets ID3 [75]. This is interesting as ID3 is also a neuronal target of MeCP2 which is the causative gene for Rett syndrome in which afflicted children often exhibit autistic-like behaviors [76]. The overlap in the clinical symptoms of ASD, ADHD, and neurodegenerative disorders raises the question of whether epigenetic regulation of ID3 plays a role.

Population-based studies have demonstrated an association between toxic environmental chemical exposure and impaired neurodevelopment that may impact neurobehavioral disorders [77]. Exposure to air pollutants from traffic and coal emissions is well-known risk factors for both attention deficit hyperactivity disorder (ADHD) and autism spectrum disorders (ASD) [78]. Polychlorinated biphenyls (PCBs) are endocrine disrupting chemicals shown to adversely affect cognitive performance. Children exposed to PCBs have shown behavioral impairments as well as significant deficits in verbal and full-scale IQ [79]. Urban areas are important regional sources of airborne PCBs and population-scale airborne exposure. Although PCBs have not been intentionally produced in the USA since the late 1970s, they continue to be detected in ambient air samples throughout the world [80]. PCBs are measurable in the blood of nearly 80% of Americans over age 50 years [81]. Hence, exposure to PCBs has been proposed to disrupt developing neuronal circuits that may cause developmental brain disorders such as learning disorders (LD), ADHD, and autism.

Endothelial cells of the blood-brain barrier (BBB) may provide clues in the study of brain health, behavior, and the environment [4]. Inhaled air pollutants can disrupt the BBB by inducing proinflammatory cytokines that act on endothelial cells [82]. We have shown evidence for how PCB-induced reactive oxygen species (ROS) may contribute to cerebral vascular phenotype changes with the goal of understanding consequences the environmental exposure has on the BBB [28]. Toxic chemical exposure can change brain gene expression through regulatory epigenetic mechanisms involving alterations in DNA methylation and histone acetylation. Evidence from animal studies show that epigenetic programming by fetal exposure to toxicants has long-lasting consequences for gene expression in the brain as well as behavior [83]. Epigenetic changes in ID3 have been associated with maltreatment of children and demonstrated as a predictor for depression [74]. Epigenetic biomarkers in peripheral tissues (blood, saliva, or buccal cells) may be useful to predict neurodevelopmental disorders in humans. Exposure to prenatal stress, famine, and pollution/toxins, factors known to affect brain development, has been associated with epigenetic variation in human peripheral tissues [84]. Based on these evidences, we postulate that ID3 can be a useful biological marker of epigenetic perturbations caused by toxic chemical exposure in children/adolescents (Figure 3(b)).

### 3.3. Kidney Disease

Lipoprotein abnormalities have been reportedly linked to renal dysfunction in chronic kidney disease patients. Nackiewicz et al. reviewed the prominent characteristics of kidney disease previously stated in ApoE^−/−^ID3^−/−^double knockout mice and show that ID3 in hyperlipidemic mice directly effects vulnerability to kidney disease. ID3 deficiency may intensify CXCL1 production by glomerular cells in response to inflammatory lipids and the resulting macrophage recruitment. Because ID3 is present in multiple cell types, it is also conceivable that other glomerular cells lacking ID3 may contribute to cytokine production in vivo [85]. Therefore, the renoprotective effect of ID3 may be through regulation of local chemokine production. ID3 is known to directly interact with more than 30 different transcription factors [86]. Noticeably, a change in the ID3 function may impact a wide range of protein-protein interactions with potentially significant consequences [87]. In dissimilarity to the findings of Nachkiewicz et al., in ApoE^−/−^ID3^−/−^ mice with glomerulonephritis, the hyperlipidemic ID3^−/−^ mice did not express meaningful increase in glomerular immune complex deposition associated with hyperlipidemic WT mice. Apolipoprotein E deficiency is known to cause enlarged immune responsiveness [88] and these results add significance to the study in dissecting the effects of ID3 alone on kidney disease.

**Figure 4:**
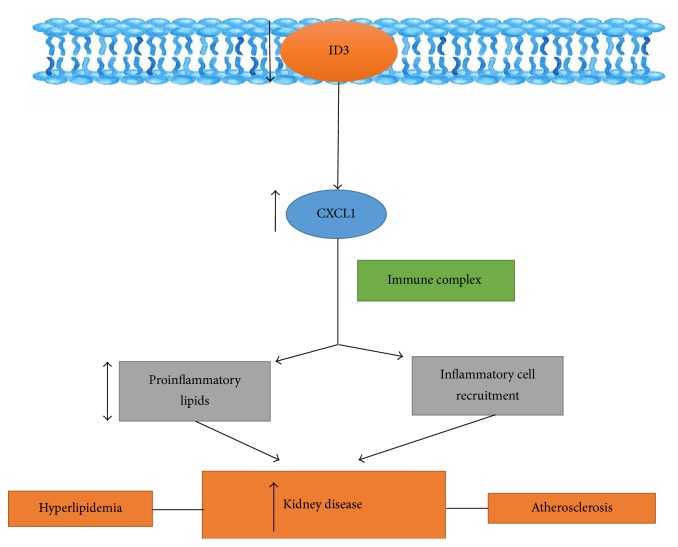
ID3 involvement in kidney disease shows a link to hyperlipidemia and atherosclerosis.

Clinical studies deliver indication for the relationship between lipids and chronic kidney disease. Nevertheless, they fail to elucidate why certain individuals (in the absence of diabetes or MetS) are probable to develop chronic kidney disease [89]. Increased susceptibility to atherosclerosis has been reported to be associated with an ID3 single nucleotide polymorphism (SNP) [65]. The overall preliminary findings in humans suggest a significant association between the same ID3 SNP and proteinuria, specifically influenced by small low density lipoproteins (*p* = 0.0024) [85]. C57BL/6 male mice on high fat diets (60% calorie from fat) develop MetS connected with obesity, elevated plasma glucose, proteinuria, and glomerulonephritis (GN) and this may be due to decrease in renal AMP activated protein kinase, a cellular energy sensor [90]. However, C57BL/6 female mice on high fat diets develop GN and proteinuria only in the absence of ID3 suggesting distinct pathogenic mechanisms between females and males. Examining molecular mechanisms in mice has recognized ID3 as a unique transcription factor that may contribute to kidney disease and provide mechanistic links between atherosclerosis, hyperlipidemia, and kidney disease in humans. A summary of ID3 pathway contributing to chronic kidney disease is shown in Figure 4.

### 3.4. Cancer

**Figure 5:**
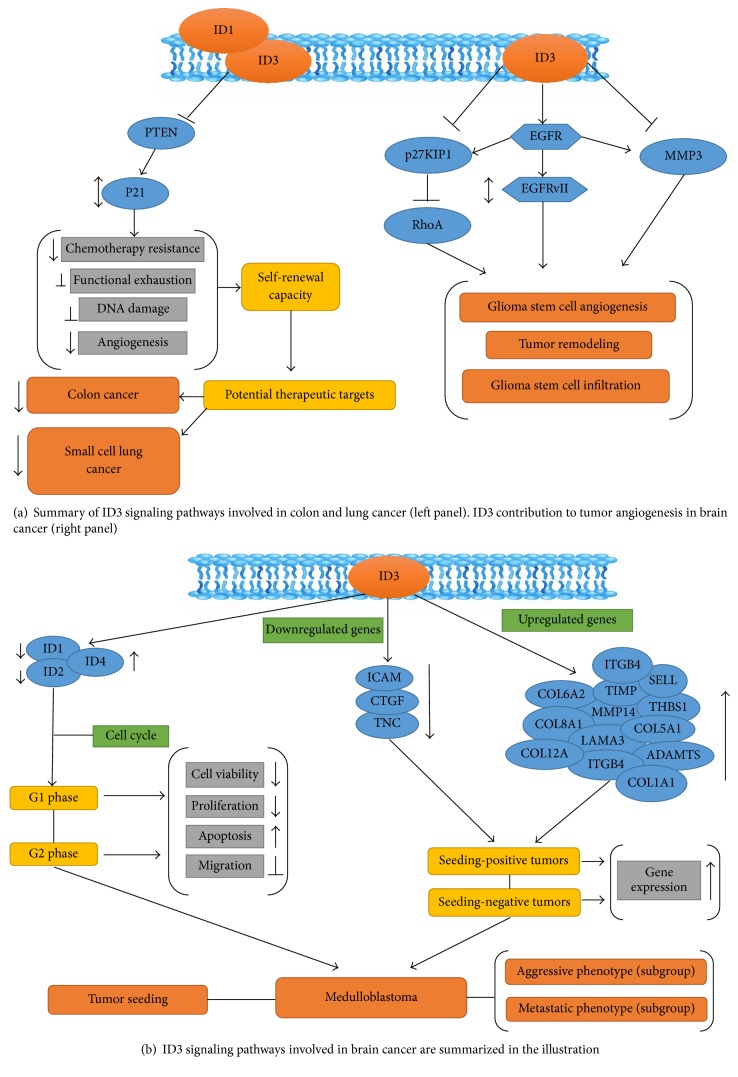


Deregulation of ID genes is reported in human cancers such as non-small cell lung cancer (NSCLC) and colon cancer. ID3 contributes in generation of hematopoietic stem and progenitor cells (HSPC) associated with myeloproliferative disease (MPD) [91]. ID1 and ID3 associated with the tumor promotion and metastasis [92]. Poor response to chemoradiotherapy has been reported in NSCLC patients with elevated ID1 and ID3 protein expression [92]. Regulation of p21 by ID1 and ID3 has been seen as the vital mechanism inhibiting the accumulation of additional DNA damage and subsequent functional low energy of colon-initiating cells (CC-ICs). Genetic silencing of ID1 and ID3 increases chemotherapy sensitivity in CC-ICs suggesting that these molecules allow cancer cells to be drug resistant [70]. Summary illustration of ID3 signaling involved in various cancer pathways is shown in Figure 5(a).

Glioblastoma multiforme (GBM) tumors contain glioma stem cells or GSCs which are implicated for glioma resistance to treatment [93–97]. ID3 is also shown to be connected with medulloblastoma in children [96]. Inhibition of ID3 reduced proliferation and migration and increased apoptosis of medulloblastoma cells. Potential molecular mechanisms of ID3 in brain cancer are shown in Figure 5(b).

### 3.5. Bone Disease

Osteogenesis imperfecta (OI) is a condition of fragile or brittle bones that break easily. OI affects 1 in 15,000 live births resulting in recurrent fractures and reduced mobility, with significant influence on quality of life [98]. BMPs (bone morphogenetic proteins) are morphogenetic signaling molecules vital for embryonic modelling. To find molecular understanding into the effect of BMPs on morphogenesis, Hollnagel et al. examined novel genes directly activated by BMP signaling. CDM- (chemically distinct growth medium-) cultured ES cells reacted very stringently to stimulation by numerous activin A, mesoderm inducers (BMP2/4), and fibroblast growth factor [99]. Using cDNA cloning, six BMP target genes were recognized. These include ID3, which exhibited convincing mRNA initiation, and the relatively stimulated Cyr61, DEK, and eIF4AII genes, as well as a gene translating a GC-binding protein. Alongside ID1, ID2, and ID3 genes were initiated by BMP4 in both ES cells and arrangement of various cell lines. ID genes encode negative regulators of basic helix-loop-helix transcription factors. In vivo, ectopic expression was observed of ID3 and Msx-2 mRNAs in Ft/1 embryos at intersecting regions of ectopic BMP4 misexpression. As a result, Hollnagel et al. proposed that the target genes of BMP4 signaling demonstrated here are part of BMP-stimulated initial processes of mammalian development. The expression arrangements of Msx-1, Msx-2, c-jun, ID1, ID2, and ID3 in normal mice versus those lacking in BMP2, BMP4, and BMP2/4-type I receptor will be of distinctive interest to compare. The promoters of the genes recognized in the analysis will aid as valuable tools to illustrate the molecular governing circuits that are overseen by BMP signaling [99].

**Figure 6:**
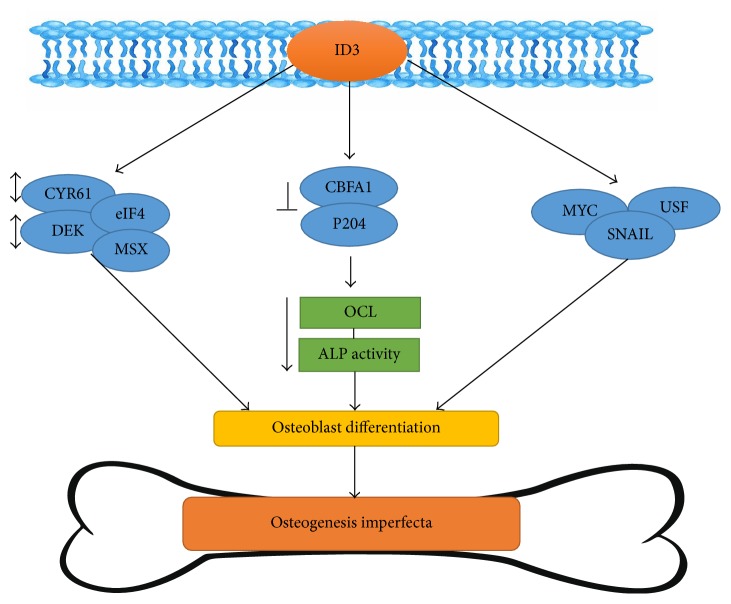
ID3 involvement in brittle bone disease pathogenesis described in the summary illustration.

ID proteins, including ID1, ID2, and ID3, are involved with essential binding factor *α*-1 (Cbfa1) to trigger debilitated transcription of the osteocalcin (OCL) and alkaline phosphatase (ALP) gene, commanding to weakened ALP action and osteocalcin (OCL) production. ID acts by hindering the specific-sequence binding of Cbfa1 to DNA and diminishing the expression of Cbfa1 in cells experiencing osteogenic differentiation [100]. Summary illustration of ID3 signaling in OI or brittle bone disease is shown in Figure 6. p204, an interferon-inducible protein that acts with both Cbfa1 and ID2, debilitated the ID2-mediated inhibition of Cbfa1-induced ALP action and OCL production. Luan et al. establish that p204 interrupted the binding of ID2 to Cbfa1 and facilitated Cbfa1 to bind to the promoters of its target genes. Furthermore, p204 stimulated the translocation from nucleus to the cytoplasm and enhanced the degradation of ID2 by ubiquitin-proteasome pathway during osteogenesis. Nucleus export signal (NES) of p204 is necessary for the p204-enhanced cytoplasmic translocation and degradation of ID2, since a p204 mutant-requiring NES lost these activities. Taken together, ID proteins help to shape a regulatory circuit and take part to control osteoblast differentiation [100].

ID1 and ID3 function to regulate bone metabolism in vivo [101]. ID1/ID3 heterozygous knockout mice showed that the thickness of calvarial junctions was attenuated by more than 50% [101]. Suppression of proliferation and mineralization in osteoblasts resultant from ID1/ID3 heterozygous knockout mice was proposed as a mechanism. Moreover, ID1/ID3 heterozygous knockout mice inhibited BMP-stimulated bone development in vivo. Hence, ID1 and ID3 are critical regulators that support bone formation in vivo [101].

The connection between mechanisms of MetS and bone mineral density (BMD) is controversial [102]. Von Muhlen et al. examined the association of MetS with osteoporosis, osteoporotic fractures, and BMD. MetS was associated with decreased, not increased BMD. Frequency of osteoporotic nonvertebral breakages was greater in members with MetS. MetS may be an additional risk factor for osteoporotic fractures [102]. Occurrence of MetS at reference was 23.5% in men and 18.2% in women. Age-adjusted analyses demonstrated in both men and women with MetS had increased BMD at total hip when compared to those without MetS (*p* < 0.001 and *p* = 0.01, resp.). Men not women with MetS furthermore had greater BMD at femoral neck (*p* = 0.05). Subsequently modifying for BMI, these connections were inverted, such that MetS was linked with decreased and not increased BMD. Occurrence of osteoporotic nonvertebral breakages was increased in participants with MetS. The connection of MetS with increased BMD was explained by the increased BMI in those with MetS [102].

### 3.6. Autoimmune Diseases

MetS has been involved in autoimmune diseases. One particular autoimmune disease is primary Sjögren's syndrome (pSS), which is primarily categorized by inflammatory association of the exocrine glands important to dry eye and mouth. Numerous organ systems can be disturbed which can cause a wide variety of extraglandular indicators, such as small airway disorders, multiple sclerosis-like disease, peripheral neuropathy glomerulonephritis, and lymphoma [101]. pSS predominately affects females (9 : 1), with a frequency in the overall population from 0.1 to 0.6%. Studies evaluating patients with rheumatoid arthritis (RA) [2] and systemic lupus erythematosus (SLE) [103] have shown that inflammation plays a role in the progression of hypertension, diabetes mellitus, and MetS [104].

The ID3 gene is involved in the growth and function of B and T cells. Deficient ID3 mice develop autoimmune disease comparable to human primary Sjögren's syndrome (pSS). Together B and T lymphocytes have been involved to contribute to the disease phenotype in this model [105]. The upregulation of ID1 and ID3 genes has been reported in patients with rheumatoid arthritis (RA) [106]. Elevated expression of ID1 and ID3 in endothelial cells has been proposed to contribute to severe angiogenesis found in RA.

## 4. Conclusion

We have comprehensively reviewed the existing evidence to illustrate the association between ID3 and metabolic perturbations. Furthermore, we extended this understanding of how ID3 and metabolic perturbations by environmental factors such as EDCs can modify chronic disease risk and health outcomes. ID3 has been seen to interact with multiple diseases such as cancer, vascular, neurological, autoimmune, and bone diseases. Epidemiological and animal model studies have shown connections between ID3 and metabolic perturbations in chronic disease. Research is warranted to better define the influence of EDCs to ID3-induced metabolic perturbations. This may lead to novel pathways for how the interaction of ID3, EDCs, and metabolic disorders exacerbates complex chronic disease and can help public health professionals control these metabolic disorders.

## Figures and Tables

**Table 1:** ID3 and endocrine disrupting chemicals (EDCs) interactions. Table created from CTD (Comparative Toxicogenomic Database).

Chemical name	Chemical ID	CAS RN	Interaction count	Organism count	Interaction	PubMed ID	Authors	Title	Year	Citation	Organism
Tetrachlorodibenzodioxin	D013749	1746-01-6	18	3	Tetrachlorodibenzodioxin affects the expression of ID3 mRNA	23238561	Nault et al.	Comparison of TCDD-elicited genome-wide hepatic gene expression in Sprague-Dawley rats and C57BL/6 mice.	2013	Toxicol Appl Pharmacol. 2013 Mar 1; 267(2): 184-91.	*Rattus norvegicus*
					Tetrachlorodibenzodioxin cotreated with TIPARP gene mutant form results in decreased expression of ID3 mRNA	21496263	Dere et al.	Differences in TCDD-elicited gene expression profiles in human HepG2, mouse Hepa1c1c7, and rat H4IIE hepatoma cells.	2011	BMC Genomics. 2011; 12: 193.	*Rattus norvegicus*
					Tetrachlorodibenzodioxin results in increased expression of ID3 mRNA	19684285	Kim et al.	Comparative analysis of AhR-mediated TCDD-elicited gene expression in human liver adult stem cells.	2009	Toxicol Sci. 2009 Nov; 112(1): 229-44.	*Homo sapiens*
						17942748	Boverhof et al.	Inhibition of estrogen-mediated uterine gene expression responses by dioxin.	2008	Mol Pharmacol. 2008 Jan; 73(1): 82-93.	*Rattus norvegicus*
						15591033	Watanabe et al.	Comparative uterine gene expression analysis after dioxin and estradiol administration.	2004	J Mol Endocrinol. 2004 Dec; 33(3): 763-71.	*Rattus norvegicus*

Bisphenol A	C006780	80-05-7	5	3	Bisphenol A results in increased expression of ID3 mRNA	26982218	Porreca et al.	“Stockpile” of slight transcriptomic changes determines the indirect genotoxicity of low-dose BPA in thyroid cells.	2016	PLoS One. 2016; 11(3): e0151618.	*Rattus norvegicus*
						25181051	Ali et al.	Exposure to low-dose bisphenol A impairs meiosis in the rat seminiferous tubule culture model: a physiotoxicogenomic approach.	2014	PLoS One. 2014; 9(9): e106245.	*Rattus norvegicus*
						25270620	Schaap et al.	A novel toxicogenomics-based approach to categorize (non)genotoxic carcinogens.	2014	Arch Toxicol. 2014 Oct 2.	*Mus musculus*
						16474171	Buterin et al.	Convergent transcriptional profiles induced by endogenous estrogen and distinct xenoestrogens in breast cancer cells.	2006	Carcinogenesis. 2006 Aug; 27(8): 1567-78.	*Homo sapiens*
						25912373	Fic et al.	Genome-wide gene expression profiling of low-dose, long-term exposure of human osteosarcoma cells to bisphenol A and its analogs bisphenols AF and S.	2015	Toxicol In Vitro. 2015 Aug; 29(5): 1060-9.	*Homo sapiens*

Ethinylestradiol	D004997	57-63-6	5	2	Ethinylestradiol inhibits the reaction [IL1B protein results in increased expression of ID3 mRNA]	12072388	Evans et al.	Estrogen receptor alpha inhibits IL-1beta induction of gene expression in the mouse liver.	2002	Endocrinology. 2002 Jul; 143(7): 2559-70.	*Mus musculus*

Benzo(a)pyrene	D001564	50-32-8	4	2	Benzo(a)pyrene results in increased expression of ID3 mRNA	22228805	Kerley-Hamilton et al.	Inherent and benzo[a]pyrene-induced differential aryl hydrocarbon receptor signaling greatly affects life span, atherosclerosis, cardiac gene expression, and body and heart growth in mice.	2012	Toxicol Sci. 2012 Apr; 126(2): 391-404.	*Mus musculus*
						20064835	Sparfel et al.	Transcriptional signature of human macrophages exposed to the environmental contaminant benzo(a)pyrene.	2010	Toxicol Sci. 2010 Apr; 114(2): 247-59.	*Homo sapiens*

Coumestrol	D003375	479-13-0	3	1	Coumestrol cotreated with 2,3-bis(3′-hydroxybenzyl)butyrolactone results in decreased expression of ID3 mRNA						
					Coumestrol cotreated with resveratrol results in decreased expression of ID3 mRNA	19167446	Dip et al.	Pleiotropic combinatorial transcriptomes of human breast cancer cells exposed to mixtures of dietary phytoestrogens.	2009	Food Chem Toxicol. 2009 Apr; 47(4): 787-95.	*Homo sapiens*
					Coumestrol results in decreased expression of ID3 mRNA						

Genistein	D019833	446-72-0	3	1							
					Genistein results in increased expression of ID3 mRNA	22228119	Di et al.	A high concentration of genistein downregulates activin A, Smad3, and other TGF-Î^2^ pathway genes in human uterine leiomyoma cells.	2012	Exp Mol Med. 2012 Apr 30; 44(4): 281-92.	*Homo sapiens*

Titanium dioxide	C009495	13463-67-7	3	2	Titanium dioxide results in increased expression of ID3 mRNA	23131501	Gao et al.	Ovarian dysfunction and gene-expressed characteristics of female mice caused by long-term exposure to titanium dioxide nanoparticles.	2012	J Hazard Mater. 2012 Dec; 243: 19-27.	*Mus musculus*

2-Amino-1-methyl-6-phenylimidazo(4,5-b)pyridine	C049584	105650-23-5	2	1	2-Amino-1-methyl-6-phenylimidazo(4,5-b)pyridine results in increased expression of ID3 mRNA	15059925	Fujiwara et al.	Global gene expression analysis of rat colon cancers induced by a food-borne carcinogen, 2-amino-1-methyl-6-phenylimidazo[4,5-b]pyridine.	2004	Carcinogenesis. 2004 Aug; 25(8): 1495-505.	*Rattus norvegicus*

Carbon tetrachloride	D002251	56-23-5	2	2	Carbon tetrachloride affects the expression of ID3 mRNA	12734012	Kriete et al.	Combined histomorphometric and gene expression profiling applied to toxicology.	2003	Genome Biol. 2003; 4(5): R32.	*Rattus norvegicus*
					Carbon tetrachloride results in increased expression of ID3 mRNA	27339419	Godoy et al.	Gene network activity in cultivated primary hepatocytes is highly similar to diseased mammalian liver tissue.	2016	Arch Toxicol. 2016 Jun 23.	*Mus musculus*

Vehicle emissions	D001335		2	2	Vehicle emissions result in increased methylation of ID3 gene	25560391	Tachibana et al.	Prenatal diesel exhaust exposure disrupts the DNA methylation profile in the brain of mouse offspring.	2015	J Toxicol Sci. 2015 Feb; 40(1): 1-11.	*Mus musculus*

1,1-Dimethylbutyl-1-deoxy-delta(9)-THC	C432747		1	1	1,1-Dimethylbutyl-1-deoxy-delta(9)-THC results in decreased expression of ID3 mRNA	15313899	Blázquez et al.	Cannabinoids inhibit the vascular endothelial growth factor pathway in gliomas.	2004	Cancer Res. 2004 Aug 15; 64(16): 5617-23.	*Mus musculus*

1,2-Dithiol-3-thione	C049325	534-25-8	1	1	1,2-Dithiol-3-thione results in decreased expression of ID3 mRNA	19162173	Ren et al.	Evidence for the involvement of xenobiotic-responsive nuclear receptors in transcriptional effects upon perfluoroalkyl acid exposure in diverse species.	2009	Reprod Toxicol. 2009 Jun; 27(3-4): 266-77.	*Rattus norvegicus*

2-(1′H-Indolo-3′-carbonyl)thiazole-4-carboxylic acid methyl ester	C548651		1	1	2-(1′H-Indolo-3′-carbonyl)thiazole-4-carboxylic acid methyl ester results in decreased expression of ID3 mRNA	19162173	Ren et al.	Evidence for the involvement of xenobiotic-responsive nuclear receptors in transcriptional effects upon perfluoroalkyl acid exposure in diverse species.	2009	Reprod Toxicol. 2009 Jun; 27(3-4): 266-77.	*Rattus norvegicus*

2,3-Bis(3′-hydroxybenzyl)butyrolactone	C029497	76543-15-2	1	1	Coumestrol cotreated with 2,3-bis(3′-hydroxybenzyl)butyrolactone results in decreased expression of ID3 mRNA	19167446	Dip et al.	Pleiotropic combinatorial transcriptomes of human breast cancer cells exposed to mixtures of dietary phytoestrogens.	2009	Food Chem Toxicol. 2009 Apr; 47(4): 787-95.	*Homo sapiens*

Diethylstilbestrol	D004054	56-53-1	1	1	Diethylstilbestrol results in decreased expression of ID3 mRNA	26865669	Ryan et al.	Moving toward integrating gene expression profiling into high-throughput testing: a gene expression biomarker accurately predicts estrogen receptor *α* modulation in a microarray compendium.	2016	Toxicol Sci. 2016 May; 151(1): 88-103.	*Homo sapiens*

Ethyl methanesulfonate	D005020	62-50-0	1	1	Ethyl methanesulfonate results in decreased expression of ID3 mRNA	24211769	Sakai et al.	Utilization of CDKN1A/p21 gene for class discrimination of DNA damage-induced clastogenicity.	2014	Toxicology. 2014 Jan 6; 315: 8-16.	*Homo sapiens*

Hexestrol	D006589	5635-50-7	1	1	Hexestrol results in decreased expression of ID3 mRNA	26865669	Ryan et al.	Moving toward integrating gene expression profiling into high-throughput testing: a gene expression biomarker accurately predicts estrogen receptor *α* modulation in a microarray compendium.	2016	Toxicol Sci. 2016 May; 151(1): 88-103.	*Homo sapiens*

Hydrogen peroxide	D006861	7722-84-1	1	1	Hydrogen peroxide affects the expression of ID3 mRNA	20044591	Briedé et al.	Global gene expression analysis reveals differences in cellular responses to hydroxyl- and superoxide anion radical-induced oxidative stress in caco-2 cells.	2010	Toxicol Sci. 2010 Apr; 114(2): 193-203.	*Homo sapiens*

Iodoacetic acid	D019807	64-69-7	1	1	Iodoacetic acid results in decreased expression of ID3 mRNA	23397585	Song et al.	Monitoring of deiodinase deficiency based on transcriptomic responses in SH-SY5Y cells.	2013	Arch Toxicol. 2013 Jun; 87(6): 1103-13.	*Homo sapiens*

Iopanoic acid	D007480	96-83-3	1	1	Iopanoic acid results in decreased expression of ID3 mRNA	23397585	Song et al.	Monitoring of deiodinase deficiency based on transcriptomic responses in SH-SY5Y cells.	2013	Arch Toxicol. 2013 Jun; 87(6): 1103-13.	*Homo sapiens*

Lead acetate	C008261	301-04-2	1	1	Lead acetate results in decreased expression of ID3 mRNA	25270620	Schaap et al.	A novel toxicogenomics-based approach to categorize (non)genotoxic carcinogens.	2014	Arch Toxicol. 2014 Oct 2.	*Mus musculus*

Lithium chloride	D018021	7447-41-8	1	1	Lithium chloride results in decreased expression of ID3 mRNA	15711924	Zhang et al.	Early gene response in lithium chloride induced apoptosis.	2005	Apoptosis. 2005 Jan; 10(1): 75-90.	*Homo sapiens*

Methoxyacetic acid	C013598	625-45-6	1	1	Methoxyacetic acid results in increased expression of ID3 mRNA	20864626	Robinson et al.	Embryotoxicant-specific transcriptomic responses in rat postimplantation whole-embryo culture.	2010	Toxicol Sci. 2010 Dec; 118(2): 675-85.	*Rattus norvegicus*

Methoxychlor	D008731	72-43-5	1	1	Methoxychlor results in decreased expression of ID3 mRNA	23397585	Song et al.	Monitoring of deiodinase deficiency based on transcriptomic responses in SH-SY5Y cells.	2013	Arch Toxicol. 2013 Jun; 87(6): 1103-13.	*Homo sapiens*

Methylcholanthrene	D008748	56-49-5	1	1	Methylcholanthrene results in increased expression of ID3 mRNA	23397585	Song et al.	Monitoring of deiodinase deficiency based on transcriptomic responses in SH-SY5Y cells.	2013	Arch Toxicol. 2013 Jun; 87(6): 1103-13.	*Homo sapiens*

Methylmercuric chloride	C004925	115-09-3	1	1	Methylmercuric chloride results in increased expression of ID3 mRNA	20864626	Robinson et al.	Embryotoxicant-specific transcriptomic responses in rat postimplantation whole-embryo culture.	2010	Toxicol Sci. 2010 Dec; 118(2): 675-85.	*Rattus norvegicus*

Methylmercury compounds	D008767		1	1	Methylmercury compounds result in increased expression of ID3 mRNA	23397585	Song et al.	Monitoring of deiodinase deficiency based on transcriptomic responses in SH-SY5Y cells.	2013	Arch Toxicol. 2013 Jun; 87(6): 1103-13.	*Homo sapiens*

Methyl methanesulfonate	D008741	66-27-3	1	1	Methyl methanesulfonate results in decreased expression of ID3 mRNA	24211769	Sakai et al.	Utilization of CDKN1A/p21 gene for class discrimination of DNA damage-induced clastogenicity.	2014	Toxicology. 2014 Jan 6; 315: 8-16.	*Homo sapiens*

Methylnitrosourea	D008770	684-93-5	1	1	Methylnitrosourea results in decreased expression of ID3 mRNA	25270620	Schaap et al.	A novel toxicogenomics-based approach to categorize (non)genotoxic carcinogens.	2014	Arch Toxicol. 2014 Oct 2.	*Mus musculus*

Monobutyl phthalate	C028577	131-70-4	1	1	Monobutyl phthalate results in increased expression of ID3 mRNA	20864626	Robinson et al.	Embryotoxicant-specific transcriptomic responses in rat postimplantation whole-embryo culture.	2010	Toxicol Sci. 2010 Dec; 118(2): 675-85.	*Rattus norvegicus*

Mustard gas	D009151	505-60-2	1	1	Mustard gas results in increased expression of ID3 mRNA	15674844	Sabourin et al.	Time- and dose-dependent analysis of gene expression using microarrays in sulfur mustard-exposed mice.	2004	J Biochem Mol Toxicol. 2004; 18(6): 300-12.	*Mus musculus*

n-Butoxyethanol	C017096	111-76-2	1	1	n-Butoxyethanol results in decreased expression of ID3 mRNA	19812364	Laifenfeld et al.	The role of hypoxia in 2-butoxyethanol-induced hemangiosarcoma.	2010	Toxicol Sci. 2010 Jan; 113(1): 254-66.	*Mus musculus*

Nickel sulfate	C029938	7786-81-4	1	1	Nickel sulfate results in increased expression of ID3 mRNA	22714537	Clancy et al.	Gene expression changes in human lung cells exposed to arsenic, chromium, nickel, or vanadium indicate the first steps in cancer.	2012	Metallomics. 2012 Aug; 4(8): 784-93.	*Homo sapiens*

Nitrosobenzylmethylamine	C014707	937-40-6	1	1	Nitrosobenzylmethylamine results in increased expression of ID3 mRNA	17616710	Reen et al.	Effects of phenylethyl isothiocyanate on early molecular events in N-nitrosomethylbenzylamine-induced cytotoxicity in rat esophagus.	2007	Cancer Res. 2007 Jul 1; 67(13): 6484-92.	*Rattus norvegicus*

N-Nitrosomorpholine	C002741	59-89-2	1	1	N-Nitrosomorpholine results in increased expression of ID3 mRNA	19716841	Oberemm et al.	Toxicogenomic analysis of N-nitrosomorpholine induced changes in rat liver: comparison of genomic and proteomic responses and anchoring to histopathological parameters.	2009	Toxicol Appl Pharmacol. 2009 Dec 1; 241(2): 230-45.	*Rattus norvegicus*

Octylmethoxycinnamate	C118580		1	1	Octylmethoxycinnamate results in decreased expression of ID3 mRNA	23397585	Song et al.	Monitoring of deiodinase deficiency based on transcriptomic responses in SH-SY5Y cells.	2013	Arch Toxicol. 2013 Jun; 87(6): 1103-13.	*Homo sapiens*

Oxophenylarsine	C029341	637-03-6	1	1	Oxophenylarsine binds to ID3 protein	23523789	Kurooka et al.	The metalloid arsenite induces nuclear export of Id3 possibly via binding to the N-terminal cysteine residues.	2013	Biochem Biophys Res Commun. 2013 Apr 19; 433(4): 579-85.	*Mus musculus*

Perfluorooctanoic acid	C023036	335-67-1	1	1	Perfluorooctanoic acid results in decreased expression of ID3 mRNA	19162173	Ren et al.	Evidence for the involvement of xenobiotic-responsive nuclear receptors in transcriptional effects upon perfluoroalkyl acid exposure in diverse species.	2009	Reprod Toxicol. 2009 Jun; 27(3-4): 266-77.	*Rattus norvegicus*

Phenol	D019800	108-95-2	1	1	Phenol results in increased expression of ID3 mRNA	17547211	Kawata et al.	Classification of heavy-metal toxicity by human DNA microarray analysis.	2007	Environ Sci Technol. 2007 May 15; 41(10): 3769-74.	*Homo sapiens*

Polychlorinated biphenyls	D011078		1	1	Polychlorinated biphenyls analog results in decreased expression of ID3 mRNA	16474171	Buterin et al.	Convergent transcriptional profiles induced by endogenous estrogen and distinct xenoestrogens in breast cancer cells.	2006	Carcinogenesis. 2006 Aug; 27(8): 1567-78.	*Homo sapiens*

Propiconazole	C045950	60207-90-1	1	1	Propiconazole results in decreased expression of ID3 mRNA	21278054	Nesnow et al.	Propiconazole induces alterations in the hepatic metabolome of mice: relevance to propiconazole-induced hepatocarcinogenesis.	2011	Toxicol Sci. 2011 Apr; 120(2): 297-309.	*Mus musculus*

Vanadyl sulfate	C034028	27774-13-6	1	1	Vanadyl sulfate results in decreased expression of ID3 mRNA	16330358	Li et al.	Discrimination of vanadium from zinc using gene profiling in human bronchial epithelial cells.	2005	Environ Health Perspect. 2005 Dec; 113(12): 1747-54.	*Homo sapiens*

Vinclozolin	C025643	50471-44-8	1	1	Vinclozolin results in increased expression of ID3 mRNA	23034163	Skinner et al.	Epigenetic transgenerational inheritance of somatic transcriptomes and epigenetic control regions.	2012	Genome Biol. 2012; 13(10): R91.	*Rattus norvegicus*

Ethylnitrosourea	D005038	759-73-9	1	1	Ethylnitrosourea results in increased expression of ID3 mRNA	15954086	Katayama et al.	Microarray analysis of genes in fetal central nervous system after ethylnitrosourea administration.	2005	Birth Defects Res B Dev Reprod Toxicol. 2005 Jun; 74(3): 255-60.	*Rattus norvegicus*

2,6-Dinitrotoluene	C023514	606-20-2	1	1	2,6-Dinitrotoluene affects the expression of ID3 mRNA	21346803	Deng et al.	Analysis of common and specific mechanisms of liver function affected by nitrotoluene compounds.	2011	PLoS One. 2011; 6(2): e14662.	*Rattus norvegicus*

3,4-Dichloroaniline	C014464	95-76-1	1	1	3,4-Dichloroaniline results in decreased expression of ID3 mRNA	24172598	Da Rocha et al.	Diuron metabolites and urothelial cytotoxicity: in vivo, in vitro, and molecular approaches.	2013	Toxicology. 2013 Dec 15; 314(2-3): 238-46.	*Homo sapiens*

4,4′-Hexafluoroisopropylidene diphenol	C583074		1	1	4,4′-Hexafluoroisopropylidene diphenol results in increased expression of ID3 mRNA	25912373	Fic et al.	Genome-wide gene expression profiling of low-dose, long-term exposure of human osteosarcoma cells to bisphenol A and its analogs bisphenols AF and S.	2015	Toxicol In Vitro. 2015 Aug; 29(5): 1060-9.	*Homo sapiens*

7,8-Dihydro-7,8-dihydroxybenzo(a)pyrene 9,10-oxide	D015123	55097-80-8	1	1	7,8-Dihydro-7,8-dihydroxybenzo(a)pyrene 9,10-oxide results in decreased expression of ID3 mRNA	19150397	Lu et al.	Early whole-genome transcriptional response induced by benzo[a]pyrene diol epoxide in a normal human cell line.	2009	Genomics. 2009 Apr; 93(4): 332-42.	*Homo sapiens*

Bis(4-hydroxyphenyl)sulfone	C543008	80-09-1	1	1	Bis(4-hydroxyphenyl)sulfone results in increased expression of ID3 mRNA	25912373	Fic et al.	Genome-wide gene expression profiling of low-dose, long-term exposure of human osteosarcoma cells to bisphenol A and its analogs bisphenols AF and S.	2015	Toxicol In Vitro. 2015 Aug; 29(5): 1060-9.	*Homo sapiens*

Bismuth tripotassium dicitrate	C002791	57644-54-9	1	1	Bismuth tripotassium dicitrate results in increased expression of ID3 mRNA	15912405	Magnusson et al.	Gene expression changes induced by bismuth in a macrophage cell line.	2005	Cell Tissue Res. 2005 Aug; 321(2): 195-210.	*Rattus norvegicus*

tert-Butylhydroperoxide	D020122	75-91-2	1	1	tert-Butylhydroperoxide results in increased expression of ID3 mRNA	15003993	Ma et al.	The effect of stress withdrawal on gene expression and certain biochemical and cell biological properties of peroxide-conditioned cell lines.	2004	FASEB J. 2004 Mar; 18(3): 480-8.	*Mus musculus*

C646 compound	C584509		1	1	C646 compound results in decreased expression of ID3 mRNA	26191083	Gaddis et al.	Altering cancer transcriptomes using epigenomic inhibitors.	2015	Epigenetics Chromatin. 2015; 8: 9.	*Homo sapiens*

Cadmium sulfate	C037123	10124-36-4	1	1	Cadmium sulfate affects the reaction [MTF1 affects the expression of ID3 mRNA]	16221973	Wimmer et al.	Two major branches of anti-cadmium defense in the mouse: MTF-1/metallothioneins and glutathione.	2005	Nucleic Acids Res. 2005; 33(18): 5715-27.	*Mus musculus*

Cobaltous chloride	C018021	7646-79-9	1	1	Cobaltous chloride results in decreased expression of ID3 mRNA	19320972	Hang et al.	Transcription and splicing regulation in human umbilical vein endothelial cells under hypoxic stress conditions by exon array.	2009	BMC Genomics. 2009; 10: 126.	*Homo sapiens*

Copper sulfate	D019327	7758-98-7	1	1	Copper sulfate results in increased expression of ID3 mRNA	19549813	Song et al.	Physiological and toxicological transcriptome changes in HepG2 cells exposed to copper.	2009	Physiol Genomics. 2009 Aug 7; 38(3): 386-401.	*Homo sapiens*

Cuprizone	D003471	370-81-0	1	1	Cuprizone affects the expression of ID3 mRNA	26577399	Abe et al.	Developmental cuprizone exposure impairs oligodendrocyte lineages differentially in cortical and white matter tissues and suppresses glutamatergic neurogenesis signals and synaptic plasticity in the hippocampal dentate gyrus of rats.	2016	Toxicol Appl Pharmacol. 2016 Jan 1; 290: 10-20.	*Rattus norvegicus*

Sodium arsenite	C017947	13768-07-5	9	2	ID3 protein inhibits the reaction [sodium arsenite results in increased expression of EGR1 mRNA]	23523789	Kurooka et al.	The metalloid arsenite induces nuclear export of Id3 possibly via binding to the N-terminal cysteine residues.	2013	Biochem Biophys Res Commun. 2013 Apr 19; 433(4): 579-85.	*Mus musculus*

Sodium arsenite					Leptomycin B inhibits the reaction [sodium arsenite affects the localization of ID3 protein]	23523789	Kurooka et al.	The metalloid arsenite induces nuclear export of Id3 possibly via binding to the N-terminal cysteine residues.	2013	Biochem Biophys Res Commun. 2013 Apr 19; 433(4): 579-85.	*Mus musculus*

Sodium arsenite					Sodium arsenite affects the localization of ID3 protein	16966095	McNeely et al.	Exit from arsenite-induced mitotic arrest is p53 dependent.	2006	Environ Health Perspect. 2006 Sep; 114(9): 1401-6.	*Homo sapiens*

Sodium arsenite					Sodium arsenite binds to ID3 protein	12760830	Andrew et al.	Genomic and proteomic profiling of responses to toxic metals in human lung cells.	2003	Environ Health Perspect. 2003 May; 111(6): 825-35.	*Homo sapiens*

Sodium arsenite					Sodium arsenite which binds to ID3 protein promotes the reaction [XPO1 protein affects the localization of ID3 protein]	23523789	Kurooka et al.	The metalloid arsenite induces nuclear export of Id3 possibly via binding to the N-terminal cysteine residues.	2013	Biochem Biophys Res Commun. 2013 Apr 19; 433(4): 579-85.	*Mus musculus*

Sodium arsenite					Sodium arsenite results in increased expression of ID3 mRNA	16966095	McNeely et al.	Exit from arsenite-induced mitotic arrest is p53 dependent.	2006	Environ Health Perspect. 2006 Sep; 114(9): 1401-6.	*Homo sapiens*

Cadmium chloride	D019256	10108-64-2	2	2	Cadmium chloride results in increased expression of ID3 mRNA	16221973	Wimmer et al.	Two major branches of anti-cadmium defense in the mouse: MTF-1/metallothioneins and glutathione.	2005	Nucleic Acids Res. 2005; 33(18): 5715-27.	*Mus musculus*

Diethylhexyl phthalate	D004051	117-81-7	2	1	Diethylhexyl phthalate results in decreased expression of ID3 mRNA	19850644	Ren et al.	Characterization of peroxisome proliferator-activated receptor alpha-independent effects of PPAR-alpha activators in the rodent liver: di-(2-ethylhexyl) phthalate also activates the constitutive-activated receptor.	2010	Toxicol Sci. 2010 Jan; 113(1): 45-59.	*Mus musculus*
